# Immunocontraceptive potential of a GnRH receptor-based fusion recombinant protein

**DOI:** 10.1186/s43141-021-00164-9

**Published:** 2021-05-04

**Authors:** Nathaniel Philip Sandam, Dhamodhar Prakash, Prashanth Thimmareddy

**Affiliations:** 1 Department of Biotechnology, M.S. Ramaiah Institute of Technology, Bangalore, India; 2 Geniron Biolabs Pvt. Ltd., Bangalore, India

**Keywords:** Dogs, Immunocontraception, GnRH, GnRHR, ZP3, Recombinant fusion protein, Contraceptive vaccine

## Abstract

**Background:**

The management of stray dog population has been of utmost importance due to their overpopulation, increase in dog bites incidence, and rabies. Contraceptive vaccines, a non-surgical alternative to spaying and neutering are viewed as a valuable option for the management of dog population. In this study, the contraceptive potential of a recombinant fusion protein containing the three genes GnRH, GnRH receptor, and ZP3 was explored.

**Results:**

The gene fragment encoding GnRH, GnRHR, and ZP3 along with the antigenic epitopes of canine distemper virus and tetanus toxoid was assembled, synthesized, and cloned into pET28a expression vector. The resulting construct GVAC08 was successfully transformed into BL21DE3 strain of *E. coli* and confirmed by colony PCR. The recombinant GVAC08 protein was expressed and purified using Ni-NTA and was confirmed to be a 50-KDa protein by SDS PAGE and Western blot. Mice were immunized with the GVAC08 protein using Freund’s complete adjuvant followed by a booster using Freund’s incomplete adjuvant. This induced a high antibody titer against GnRH, GnRH receptor, and ZP3 which was determined by ELISA.

**Conclusion:**

Mating studies showed that the GVAC08 recombinant protein was able to reduce the litter size in immunized mice showing improved efficacy. However, the vaccine candidate with further improvements will be a viable contraceptive vaccine.

## Background

The ever-increasing population of stray dogs in India and around the world has become a threat to society. In 2012, the dog population was estimated to be around 525 million and by 2018 it was estimated to be 700 million out of which 75–80% of dogs are strays also called as free-ranging dogs [1, 2]. According to WHO Expert consultation on rabies, 99% of human deaths due to rabies is caused by dogs [3, 4].

Stray dogs pose a serious threat as they are often carriers of the rabies virus and can infect humans when they bite them [5]. These bites can be fatal even if it does not transmit the virus. Stray dogs cause disease transmission, predation, attacks on humans, and road accidents and also cause nuisance [6, 7].

Hence, there is a need to control the stray dog population. Currently, the only solution to control stray dog population is sterilization. The most common form of sterilization is spaying of females and surgical castration of males. However, surgical sterilization is expensive and time consuming, has longer recovery time [8], and requires the expertise of a veterinary surgeon. Hence, there is a need for a more convenient, cost effective, easy to use, non-surgical method of sterilization. The answer to this is to use a vaccine that can lead to sterility; this method is called as immunocontraception.

The need for contraceptive medications for animals started doing the rounds in the 1960s–1970s. Studies in progestin-based contraceptives started in Europe in 1963; however, since the effects of progestins have shown varying results and acceptance by veterinarians, they have not taken off [9]. The use of progestin-based contraceptives increases the risk of cardiovascular diseases and higher doses of progestin increases risk of breast cancer [10].

Hormonal antigens are a target for immunocontraception. The antigenicity of Gonadotropin-releasing hormone (GnRH) had been confirmed in the 1970s. Since it is a small molecule, it needs to be conjugated to larger proteins or potent yet safe adjuvants.

The main targets for immunocontraception have been GnRH, zona pellucida (ZP), and more recently GnRH receptors. GnRH hormone is synthesized by the hypothalamus and regulates the secretion of luteinizing hormone (LH) and follicle-stimulating hormone (FSH) which are hormones essential for reproduction [11].

The hypothalamus produces gonadotropin-releasing hormone (GnRH) and secretes it into the blood stream; the GnRH signals are received by the anterior pituitary gland and secrete FSH which in turn stimulates LH causing follicular growth and maturation and induces ovulation [12]. The immune system produces anti-GnRH antibodies when vaccinated against GnRH; these bind to circulating GnRH in the blood stream and blocks GnRH activity. This results in reduction of FSH and LH production by the anterior pituitary gland leading to reduction in ovarian follicular development which results in insufficient estrogen levels to cause behavioral estrus [13].

GnRH-based contraceptive vaccines have been used in the management of wildlife species, one such vaccine is the Gonacon (USDA, Pocatello, ID, USA) which has been effective in many animal species [14–16]. Zona pellucida glycoprotein-based contraceptive vaccines are in use to control wildlife population of feral horses and white-tailed deer and kangaroos [17–22].

ZP plays an important role in the fertilization process. ZP3 glycoprotein is recognized by the sperm and the sperm binds to the ZP3 glycoprotein which also causes the acrosome reaction of the sperm and hence is a target for contraception [23, 24]. Several studies show that ZP3 has the ability to reduce the population in mice [25–28].

In the present study, GnRH, GnRH receptor (GnRHR), and ZP3 have been cloned into pET 28a expression system for the first time with the objective to improve the efficacy of the contraceptive vaccine. The cloning of all three genes to produce one recombinant protein is being reported for the first time. The resultant protein GVAC08 was purified and immunized in mice to check for its contraceptive effect. The results of which will throw some light towards hurdles in contraceptive vaccine production.

## Methods

### Cloning of GnRH, GnRH receptor, and ZP3 into pET 28a vector

The sequence of GnRH, GnRH receptor, and ZP3 were retrieved from NCBI and were assembled along with the antigenic epitopes of canine distemper virus and tetanus toxoid. The length of the sequence was 1760 base pairs. This sequence was synthesized and cloned between NCO I and Xho I restriction sites using the primers V04FP 5′CCATGGGGATGGCGAGCGCGAG3′ and V08RP 5′CTCGAGGCTCAGGCTCGGGGTACCG 3′ as mentioned in Table 1. Using standard protocols, the synthesized gene sequence and the pET 28a vector were digested with NCO I and XHO I restriction endonucleases and cleaned using Ultra PCR clean up kit (Thermo Scientific). The digested vector and gene were ligated with T4 DNA ligase resulting in GVAC08 plasmid. The plasmid was subjected to double digestion with NCOI and XhoI followed by Agarose gel electrophoresis of digested plasmid to confirm an insert size of 1760 base pairs.

**Table 1:** Primers used for cloning and PCR

Gene construct	Primer name	Primer sequence 5′-3′
GVAC08	V04FP	CCATGGGGATGGCGAGCGCGAG
	V08RP	CTCGAGGCTCAGGCTCGGGGTACCG
	T7 forward	TAATACGACTCACTATAGGG
	T7 reverse	GCTAGTTATTGCTCAGCGG

### Protein expression and purification

The plasmid was transformed into BL21 DE3 *E. coli* strain and the transformed cells were plated on LB plates containing 50 μg/ml kanamycin and then incubated at 37 °C. The resulting colonies were sub-cultured.

Transformation of BL21 DE3 cells containing GVAC08 plasmid was confirmed by colony PCR using T7 forward and reverse primers; briefly, PCR master mix was prepared along with the mentioned primers and aliquoted into several tubes. Using a sterile toothpick, small portions of the colonies were picked and mixed along with the tubes containing the master mix and PCR carried out. Care was taken to prevent cross contamination of different colonies in the tubes. The tubes were then run on an agarose gel electrophoresis. A positive clone was inoculated in 10 ml LB broth containing 50 μg/ml kanamycin and incubated at 37 °C overnight.

Overnight grown cells were inoculated into 500 ml LB broth and incubated at 37 °C for 3.5 h, i.e., till the OD_600_ 0.6 was reached. The cells were then induced with IPTG (isopropyl β-d-1-thiogalactopyranoside) to a final concentration of 1 mM and further incubated at 37 °C for 6 h for the expression of recombinant GVAC08 protein.

The culture was centrifuged at 6000 rpm for 20 min and the pellet re-suspended in 20 ml of lysis buffer (8 M urea, 50 mM Na_2_HPO_4_, 300 mM NaCl, 1 mM PMSF, pH 8). The cells were lysed by sonication on ice 37% amplitude and 10-s burst cycle for 15 min. The sonicated mixture was centrifuged at 10,000 rpm for 10 min. The supernatant was collected and then added into a column containing 2 ml Ni NTA slurry and mixed gently on a cyclo mixer for 1 h at 4 °C. The column was washed with wash buffer containing 20 mM imidazole. The GVAC08 protein was eluted with elution buffer containing 300 mM imidazole. The eluted fractions were pooled and transferred into a 10-KDa snakeskin dialysis bag (Thermo Scientific). Dialysis was carried out in a step wise manner each time reducing the concentration of dialysis buffer from 7 M urea to 0 M urea and finally in 1X PBS.

The purity of the protein was assessed on a 10% SDS PAGE gel and confirmed by Western blot using anti-His monoclonal antibody as the primary antibody and goat anti-mouse IgG-HRP as the secondary antibody, and the membrane was developed using DAB substrate. The protein concentration was estimated using BCA Kit (Pierce).

### Immunization of mice

All animal studies were undertaken as per the Committee for the Purpose of Control and Supervision of Experiments on Animals guidelines (*CPCSEA No. 493/GO/ReBiBt-S/Re-L/01*) after the necessary approvals.

Six to 8-week-old Balb/c mice with a body weight of about 25–30 g obtained from Geniron Biolabs Pvt. Ltd. Bangalore, India were used for the study. The mice were randomly grouped into three groups, group I, group II, and group III; each group had 12 mice—6 male and 6 female mice. The six males and six females were housed separately. Group I mice were immunized individually with 50 μg GVAC08 recombinant protein, group II were immunized with 100 μg GVAC08 recombinant protein, and group III immunized with saline served as control. Group I and group II male and female Balb/c mice were immunized with 50 μg and 100 μg GVAC08 protein as mentioned in Table 2, and mixed with Freund’s complete adjuvant (Sigma Aldrich) by the intradermal route and a booster injection was given with Freund’s incomplete adjuvant (Sigma Aldrich) on day 21 post primary injection. The animals were housed and maintained in controlled environment having proper temperature, light, humidity, and sound levels. The mice were checked twice daily for possible adverse reaction after immunization.

**Table 2:** Experimental design

Group	Dose	Method of administration
Group I	50 μg GVAC08	Intradermal
Group II	100 μg GVAC08	Intradermal
Group III (control)	100 μl Saline	Intradermal

### Humoral immune response

Mice were first anesthetized with 3% isoflurane according to ethics committee guideline. Blood was then collected through retro-orbital bleeding on the 0th, 14th, 28th, and 45th day post-immunization to check for antibody titers by indirect ELISA.

### Elisa

The serum antibody titer against recombinant GVAC08 containing GnRH, GnRHR, and ZP3 was determined by ELISA. Briefly, microtiter ELISA plates were coated with 200 ng/well of GVAC08 protein and incubated overnight at 4 °C. The plates were washed 3 times with PBS containing 0.05% tween 20, excess buffer removed, and 3% BSA in PBS added to block non-specific binding. The plates were washed 3 times with PBS containing 0.05% tween 20. Serum collected from the immunized mice was diluted and added to the wells and incubated for 1.5 h. The plates were washed 3 times with PBS containing 0.05% tween 20. Anti-mouse IgG conjugated to peroxidase (Jackson Immunoresearch Laboratories) was diluted to 1:50,000 in PBS and added to the wells and incubated at 37 °C for 1 h. The wells were washed and TMB substrate was added. The reaction was stopped with 1N H_2_SO_4_ and the absorbance read at 450 nm using Epoch microtiter plate spectrophotometer (Biotek Instruments).

### Mating studies

Forty-seven days post primary immunization, each immunized male was co-habitated with one unimmunized female with proven fertility separately and one female immunized mouse was co-habitated with unimmunized male mice with proven fertility separately for 30 days.

The mice were monitored daily for signs of pregnancy (enlarged abdomen and presence of fetuses detected by palpation), female mice were observed for vaginal plug formation which is an indication of mating, and once observed the male mice were removed and housed separately and the female mice were housed separately. The female mice were further observed for the number of pups delivered by mice of immunized and control groups, and the mean number of pups was calculated along with standard deviation. The immunized mice were euthanized by cervical dislocation, whereas the pups were allowed to live their natural lives.

## Results

### Cloning of GnRH, GnRH receptor, and ZP3 into pET 28a vector

**Fig. 1:**
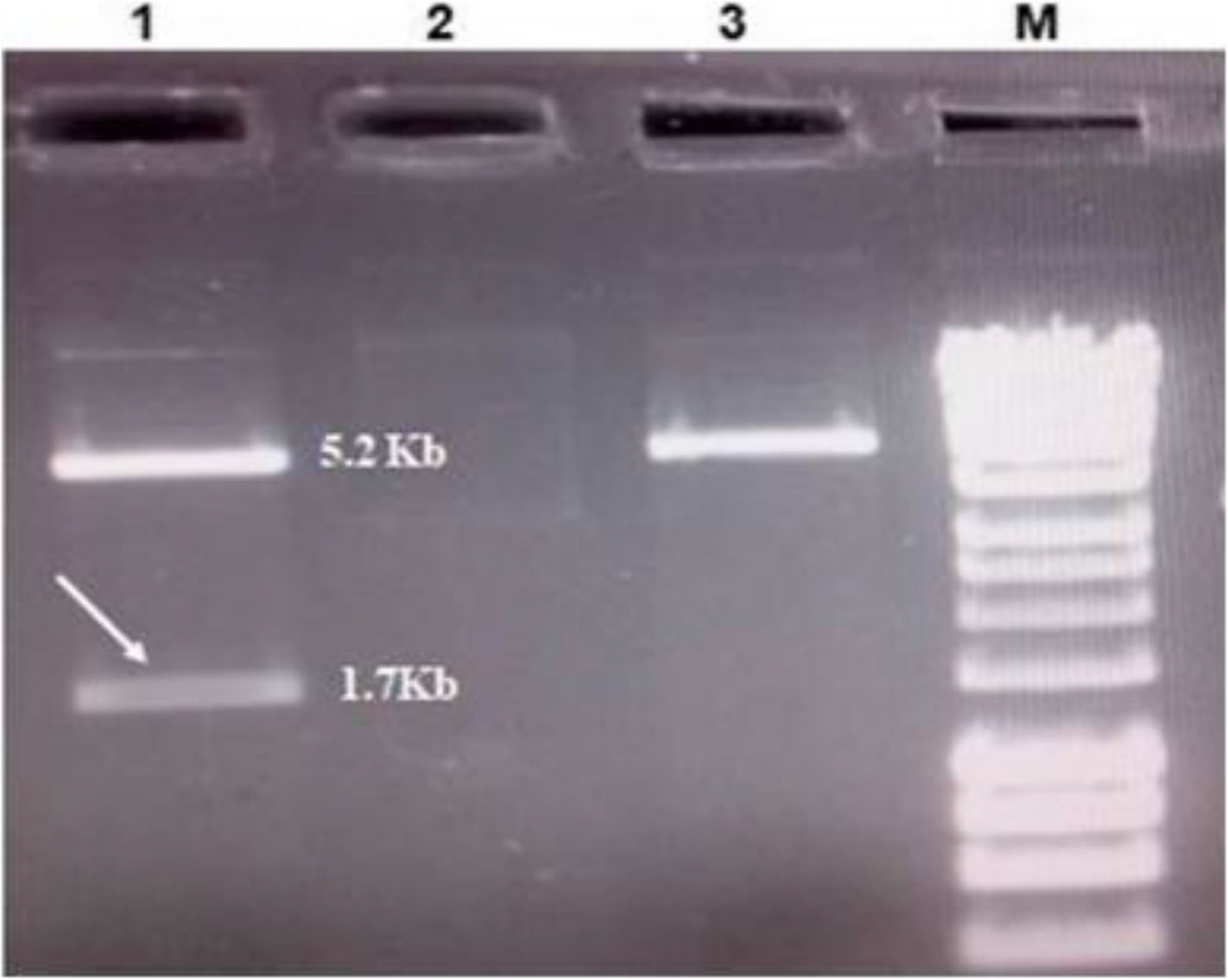
Restriction digestion of GVAC08. Lane 1, digested GVAC08; lane 3, pET28a vector; lane M, 1-Kb marker

The gene fragment encoding GnRH, GnRHR, and ZP3 along with the antigenic epitopes of canine distemper virus and tetanus toxoid was assembled, synthesized, and cloned into pET28a expression vector successfully between NcoI and XhoI restriction sites and confirmed by double digestion using the two restriction enzymes. Digestion confirmed an insert size of 1760 base pairs (Fig. 1).

### Protein expression and purification

**Fig. 2:**
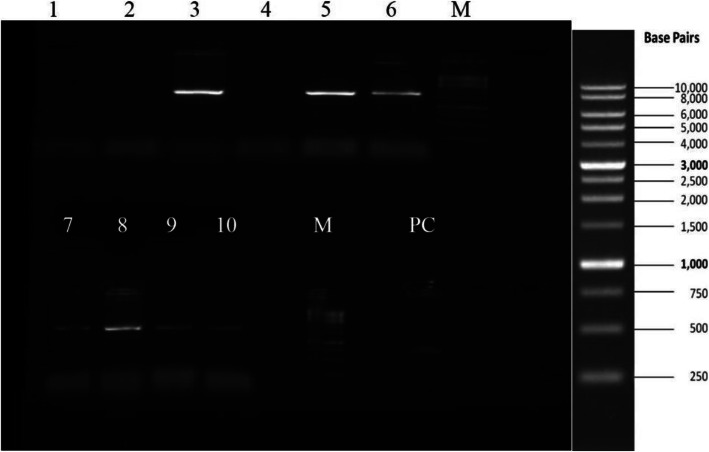
Colony PCR confirmation of transformed colonies. Lanes 1, 2, and 4, untransformed colonies; lanes 3 and 5 to 10, transformed colonies; M, marker; PC, positive control

**Fig. 3:**
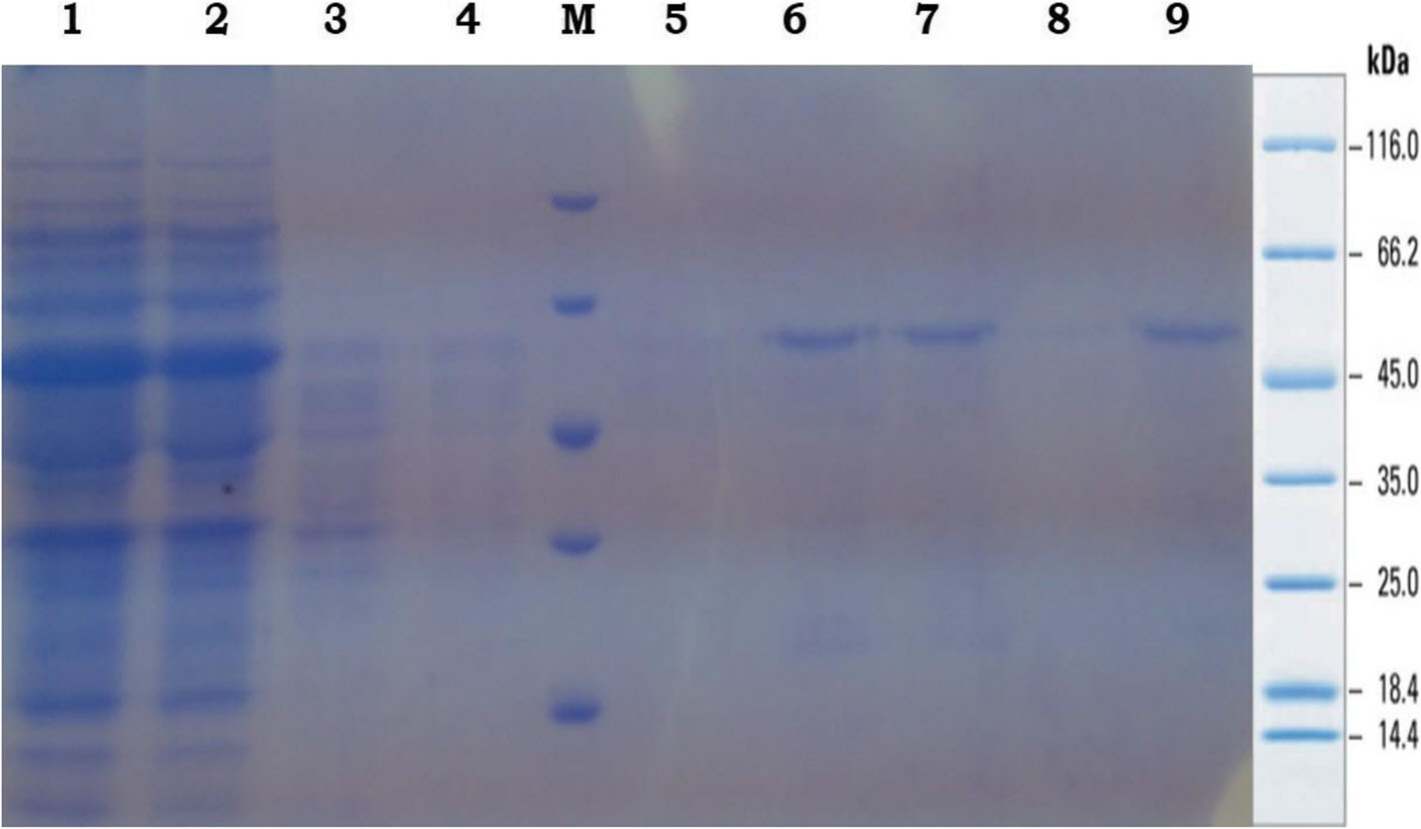
SDS PAGE analysis of recombinant GVAC08 protein (purified). Lane 1, flow through; lane 2, 3, and 4, wash elutes; lane M, unstained protein ladder Thermo Scientific; lane 5–9, elution 300 mM imidazole conc.

**Fig. 4:**
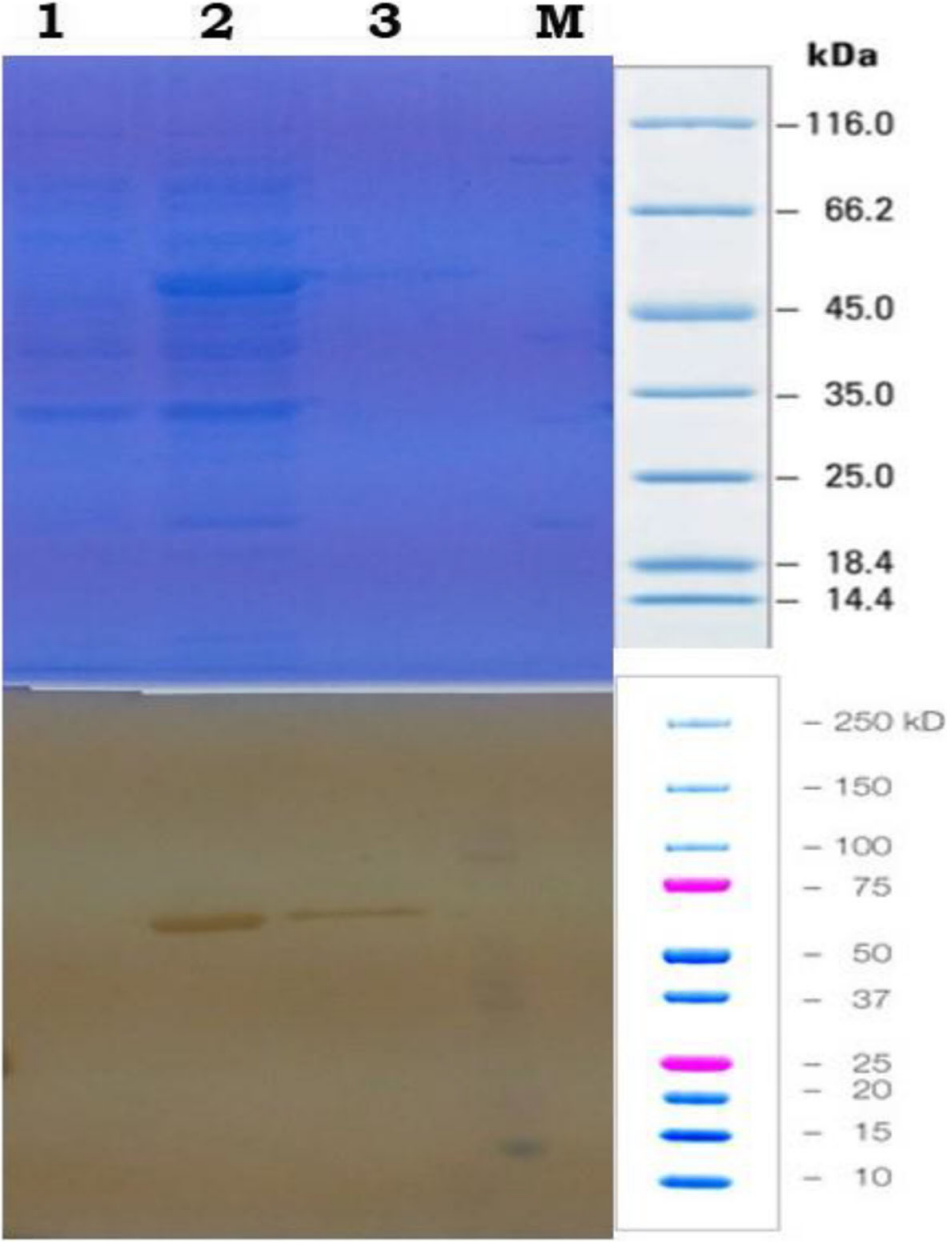
Western blot analysis of GVAC08 recombinant protein. Lane 1, uninduced BL21DE3; lane 2, IPTG induced showing GVAC08 recombinant protein at 50 kDA; lane 3, purified GVAC08 recombinant protein; lane 4A, unstained protein ladder Thermo Scientific and Precision plus dual colour protein standard (Biorad)

The gene construct GVAC08 was successfully transformed into BL21DE3 strain of *E. coli*. Transformation was confirmed by colony PCR using T7 forward and reverse primers as observed by a 1.7-kb band which corresponds to the size of the GVAC08 gene insert (Fig. 2). A 50-KDa recombinant protein was expressed with 1-mM IPTG after 6 h which corresponds to the size of GVAC08 protein. Peak expression was observed after 4 h. The cells were sonicated and clarified by centrifugation and the resulting lysate was added onto Ni-NTA slurry and the protein was eluted with 300-Mm imidazole. The eluted fractions were pooled and desalted by dialysis. A small fraction was collected for SDS PAGE (Fig. 3) and Western blot analysis (Fig. 4). SDS PAGE analysis of the purified recombinant GVAC08 protein revealed a single band corresponding to 50-kDa protein. The protein was found to be 95% pure. One liter of culture yielded 15 mg of purified GVAC08 recombinant protein.

### Immunization of mice

Mice were immunized with GVAC08 recombinant protein along with FCA followed by a booster dose with GVAC08 recombinant protein along with FIA and observed for physical and behavioral changes.

### Humoral immune response

Mice blood was collected and tested by indirect ELISA the results of which are mentioned in Table 3. The anti-GVAC08 antibody titers was estimated to be 1:8000 on the 14th day post-immunization and 1:32,000 on the 28th day which suggests that the antibody titer increased after the administration of the booster dose. Mice with high anti-GVAC08 antibody titer were used for mating to check for contraceptive effect of GVAC08 protein.

**Table 3:** Immunogenicity efficacy of recombinant GVAC08 in immunized mice

Group	Immunogen	Antigen used for ELISA	Antibody titer (× 10^3^)			
			Day 0	Day 14	Day 28	Day 45
Group I	GVAC08	GVAC08	0.055	8	28	32
Group II	GVAC08	GVAC08	0.043	8	32	45

### Mating studies

The formation of vaginal plugs was observed in 100% of control and immunized groups which indicate mating has taken place. Immunization of mice with GVAC08 recombinant protein led to a decrease in the number of pups produced in contrary to mice immunized with saline. The mean litter size of the control group was found to be 13 ± 0.5, whereas the mean litter size of the immunized group was calculated to be 9 ± 0.31.

## Discussion

Dog population control as well as other animal population control has been of utmost importance and over the past few years the importance to develop an immunocontraceptive vaccine has increased [29]. An immunocontraceptive vaccine generates antibodies against proteins involved with the reproductive system, GnRH being one of them. GnRH is a hormone secreted by the hypothalamus which in turn aides in the secretion of FSH and LH which are the important reproductive hormones. The antibodies generated prevent the normal action of GnRH by binding to the GnRH receptors and preventing the binding the GnRH hormone and hence also preventing the secretion of FSH and LH [30]. On the other hand, ZP3 is a very important glycoprotein required for the fertilization process. Therefore, GnRH and ZP3 have been the main targets of contraception for a long time; GnRHR on the other hand is a fairly new target for immunocontraception. GnRH and GnRHR are very weak immunogens owing to their small size. Therefore, several approaches have been made to increase the immunogenicity. Some of the methods used are by using multiple copies of GnRH arranged linearly, and use of toxin receptors such as tetanus toxoid [27]. The use of diphtheria toxin conjugated to zona pellucida protein has shown to improve the in vitro contraceptive efficacy, this suggests that the use of B cell epitopes of particular zona proteins may enhance contraceptive efficacy [31, 32].

In this study, the contraceptive potential of a recombinant fusion protein GVAC08 containing the three genes GnRH, GnRH receptor, and ZP3 was explored. As per our knowledge, the use of GnRH, GnRHR, and ZP in a single vaccine construct (GVAC08) is being reported for the first time. The advantage of using all 3 genes in a single construct is to increase the contraceptive effect of the vaccine. In addition, the gene fragment encoding GnRH, GnRHR, and ZP3 along with the antigenic epitopes of canine distemper virus and tetanus toxoid was assembled, synthesized, and cloned into pET28a expression vector. The use of T cell epitopes to increase a stronger anti-GnRH antibody response was in agreement with the earlier studies [33]. pET28a vector, as used in this study, has been used to express GnRH peptides [34].

Our study showed that the recombinant protein vaccine GVAC08 elicited a strong immune response generating anti-GnRH, anti-GnRHR, and anti-ZP antibodies; this resulted in the decrease of the mean litter size when compared to the control group. It was also observed that a decrease in litter size was seen when an immunized mice was mated with an unimmunized pair. This can prove beneficial as in an immunization program, it might not be possible to immunize every single dog be it a female or a male. Hence, GVAC08 recombinant protein has a huge potential in reducing dog population. Several recombinant proteins and synthetic peptides have been designed using ZP3, GnRH, and spermatozoa proteins believing that the antibodies generated by these proteins will be effective in achieving contraception [35, 36]. However, these studies have proven that the use of multiple antigens can be effective in achieving contraception. However, the results obtained so far have failed to prove its efficacy. The reason could be that antibody titer peak during a time period and then subside and eventually become undetected in the blood. With respect to contraceptive vaccine, the biggest challenge is to improve the efficacy to inhibit fertility [32].

In summary, we have developed a protein vaccine candidate targeting GnRH, GnRHR, and ZP by expressing the respective proteins under the control of T7 promoter to increase the protein expression. The construction of the fusion protein using pET28a vector allows us to maintain a high copy number of the resultant plasmid in *E. coli*. The GVAC08 recombinant fusion protein showed significant contraceptive efficacy in mice immunized with it and hence a decrease in mean litter size of immunized mice was observed when compared to the control group. However, further studies with increased dose of GVAC08 recombinant protein is required to study the effect of the GVAC08 vaccine on the reproductive organs to get a better understanding of its effect which will further allow us to improve the vaccine to achieve complete contraception.

## Conclusion

In this study, a protein-based vaccine GVAC08 directed against GnRH, GnRH receptor, and zona pellucida was developed. The vaccine candidate induced a good antibody response and a significant contraceptive effect. The mean litter size of mice immunized with the vaccine candidate was found to be lower than the control mice. Further studies need to be carried out to achieve complete contraception. With additional improvements in vaccine formulation, dose, and delivery, the GVAC08 vaccine can be an efficacious vaccine for dog population management.

## Data Availability

Authors declare that all generated and analyzed data are included in the article.

## Abbreviations

WHO: World Health Organization
GnRH: Gonadotropin-releasing hormone
ZP: Zona pellucida
GnRHR: Gonadotropin-releasing hormone receptor
LH: Luteinizing hormone
FSH: Follicle-stimulating hormone
USDA: US Department of Agriculture
NCBI: National Centre for Biotechnology Information
LB: Luria Bertani
OD600: Optical density
IgG: Immunoglobulin G
HRP: Horseradish peroxidase
DAB: 3,3-Diaminobenzidine
BCA: Bicinchoninic acid
CPCSEA: Committee for the Purpose of Control And Supervision of Experiments on Animals
ELISA: Enzyme-linked immunosorbent assay
BSA: Bovine serum albumin
Na_2_HPO_4_: Disodium hydrogen phosphate
NaCl: Sodium chloride
PMSF: Phenylmethylsulfonyl fluoride
pH: Power of hydrogen
Ni NTA: Nickle-nitrilotriacetic acid
PBS: Phosphate-buffered saline
SDS PAGE: Sodium dodecyl sulfate polyacrylamide gel electrophoresis
H_2_SO_4_: Sulphuric acid
N: Normal
PCR: Polymerase chain reaction
IPTG: Isopropyl β-d-1-thiogalactopyranoside
FCA: Freund’s complete adjuvant
FIA: Freund’s incomplete adjuvant
TMB: 3,3′,5,5′-Tetramethylbenzidine
